# Proline Accumulation Influenced by Osmotic Stress in Arbuscular Mycorrhizal Symbiotic Plants

**DOI:** 10.3389/fmicb.2018.02525

**Published:** 2018-10-29

**Authors:** Se Chul Chun, Manivannan Paramasivan, Murugesan Chandrasekaran

**Affiliations:** ^1^Department of Bioresource and Food Science, Konkuk University, Seoul, South Korea; ^2^Department of Microbiology, Bharathidasan University, Tiruchirappalli, India; ^3^Department of Food Science and Biotechnology, Sejong University, Seoul, South Korea

**Keywords:** arbuscular mycorrhiza, drought, salinity, osmolytes, proline accumulation, stress evasion

## Abstract

Salinity and drought are the major osmotic stress limitations that affect plant growth and crop yield in agriculture worldwide. The alternative response mediated by plants in response to salinity and drought are principally proline accumulation which regulates stress combat strategies owing to sustainable production in the realm of agricultural production even under severe stress. Symbiotic and soil associated arbuscular mycorrhizal fungi (AMF) are regarded as efficient biofertilizers in several crops under these stresses. Summarily AMF is renowned for effective scavengers of free radicals in soil thereby increasing soil parameters optimal for plant growth. AMF contribute to augment host plant tolerance to stress specifically salinity and drought. Mycorrhizal colonization positively regulates root uptake of available nutrients and enhance growth even when bestowed by water constraints which has contributory roles due to proline accumulation providing several intriguing researches on AMF symbiosis pertaining to plant productivity and yield. Mycorrhizal plants and their non-mycorrhizal counterparts show varied expression pattern regarding proline amass. Hence, the precise role of proline with respect to stress tolerance and equivocal mechanisms involved in evasion of osmotic stress has not been extensively reviewed earlier. Further molecular forecasting in this arena is still an underexploited research field. This review comprehensively addresses the observable facts pertaining to proline accumulation upon AMF association and adherence relevant to stress tolerance and host plant efficiency and efficacy.

## Introduction

Abiotic stress that limits plant growth and development are largely confined to salinity and drought in the realm of agriculture (Chandrasekaran et al., 2014; Augé et al., 2015). Osmotic factors owing to salinity and drought create rampage adversities upon plant production and productivity due to water constraints. Water being a universal solvent has copious optimal effects in the field of crop production and improvement strategies. Inadequate water supply poses serious threats to plants through a plethora of plant physiological parameters such as stomatal closure, reduction in gaseous exchange and chlorophyll content. This can be corroborated by drought stress even in woody plants like *Amorpha fruticosa* and *Robinia pseudoacacia* wherein stomatal closure delineates leaf and water potential based on non-hydraulic factors (Yan et al., 2017). Plants combat osmotic stress employing various coherent phenomena relating plant anatomy and physiology with cellular mechanisms (Bray, 1997; Evelin et al., 2009). Proline has been addressed as a unique low molecular weight osmolyte which response to stresses related to osmosis in wide plant varieties (Delauney and Verma, 1993; Hasegawa et al., 2000).

**FIGURE 1:**
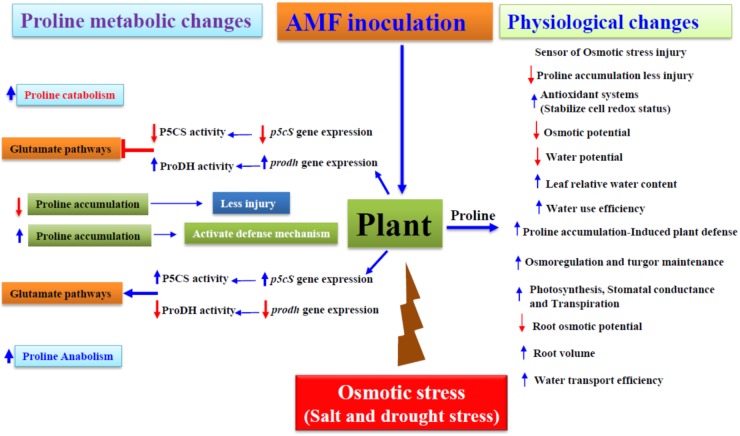
Proline accumulation influenced physiological and molecular changes by osmotic stress in Arbuscular Mycorrhizal symbiotic plants. P5CS-Δ1, pyrroline-5-carboxylate synthetase; ProDH, proline dehydrogenase. The blue arrows indicate increase/up-regulation, while the red arrows indicate decrease/down-regulation. 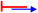, inhibition and induction, respectively.

The amino acid, proline accrues during water constraints (Hare et al., 1998), salinity (Munns, 2005), low temperature (Naidu et al., 1991), heavy metal accumulation (Sharma and Dietz, 2006) among others. Proline further is an important variable amino acid in determining protein and membrane structures and scavenge reactive oxygen species (ROS) under drought stress (Ashraf and Foolad, 2007). Proline not only act as an osmotolerant, also act as a nutritional source. Fungi utilize proline as both a nitrogen and a carbon source. *Aspergillus nidulans* relies on proline as both a nitrogen and a carbon source (Keller and Hohn, 1997) whereas, in *Saccharomyces cerevisiae*, proline serves as a nitrogen source but not a carbon source (Huang and Brandriss, 2000). Also, in endophyte, *Acremonium coenophialum* proline act as a good nitrogen source (Kulkarni and Nielsen, 1986). In this regard, proline, tyrosine, and methionine act as extremely poor sources of N for ectomycorrhizal fungi (Abuzinadah and Read, 1988). Differences in utilization of proline showed that the fungi possess a number of permeases for the uptake of amino acids, which can serve as nitrogen and/or carbon source or as building blocks for protein and peptide synthesis (Horák, 1986). Moreover, in the natural environment amino acids are normally occur in mixtures in which some of the individual components may have stimulatory, which are readily used by mycorrhizal fungi (present in ‘free’ form in soils) while others have inhibitory effects upon growth of the fungi (Abuzinadah and Read, 1988). Lack of amino acid utilization, therefore, could be due to either lack of uptake or absence of one or more enzymes in the catabolic pathway (Kulkarni and Nielsen, 1986). Researchers reported that proline utilization pathway genes are essential for the organization and regulation of the genes involved in L-proline catabolism (Keller and Hohn, 1997). Proline dehydrogenase (ProDH) and Δ^1^-pyrroline-5-carboxylate dehydrogenase (P5CDH) are the two key enzymes in the catabolism of proline. Unlike the limited studies in fungi, proline dehydrogenases have been extensively studied in plants. In *Arabidopsis thaliana*, two *ProDH* genes have been identified and functionally characterized. *ProDH1* is a dehydration-responsive gene and is up-regulated after rehydration, accompanied by a decrease of intracellular proline (Kiyosue et al., 1996). *ProDH1* appears to be the dominant isoform under most conditions and in most tissues, whereas *ProDH2* is specifically up-regulated during salt stress (Funck et al., 2010). Proline function to protect plants from drought and salinity stress (Nanjo et al., 1999), and ProDH is one of the key enzymes that regulate proline accumulation *in vivo* (Peng et al., 1996). The significant association between proline accumulation and osmotic stress tolerance has been extensively reported earlier (Delauney and Verma, 1993; Hong et al., 2000; Kishor et al., 2005). Nevertheless, proline accumulation alone cannot be correlated with osmotic stress tolerance in plants. Combinatorial effects of salinity and drought stress are linked to less water availability for plants (Augé, 2001; Parniske, 2008; Porcel et al., 2012) (Figure 1). Hitherto, it is evident that the mutualistic fungi in symbiotic plants have contributed to induce plant physiology mechanisms to assuage stress rapidly than the non-symbiotic counterparts (Rodriguez et al., 2004). Arbuscular mycorrhizal fungi (hereafter mentioned as AMF) are regarded as oldest obligate symbionts having their identity aging 400 million years of nearly 80% of terrestrial plant order colonizing root cortex biotrophically. AM (hereafter abbreviation for symbiosis) symbiosis helps plants to absorb water and mineral nutrients efficiently from the soil through extra-metrical mycelium development (García and Mendoza, 2008; Bonfante and Genre, 2010; Lee et al., 2013; Rillig et al., 2015). AM symbiosis playing pivotal roles in induced and increased stress tolerance have been explicitly studied earlier (García and Mendoza, 2008; Porcel et al., 2012; Chandrasekaran et al., 2014; Augé et al., 2015).

## Amf Mediated Alleviation of Salt Stress

It has been estimated earlier that approximately 1/3 of the farmlands on earth are prone to salt stress and salinity setback (Munns and Tester, 2008). Further, there is a dire need to deal with salinity complications as it has been expected that 30% of the cultivable land might be rendered unusable by 2050 indirectly leading to declining in sustainable food production technologies (Wang et al., 2003). Hence probing for alternate strategies for escalating plant productivity reveal that symbiosis between plant roots and AMF is one among the comprehensive strategies for alleviating salt stress (García and Mendoza, 2008; Porcel et al., 2012; Chandrasekaran et al., 2014). Mycorrhizal colonization also has been an affirmative phenomenon in increasing nutrient uptake (Porras-Soriano et al., 2009; Hajiboland et al., 2010; Evelin et al., 2012), and maintaining ionic balance (Giri et al., 2007; Evelin et al., 2012; Wu et al., 2013). The above-said fact has been proved when mycorrhizal plants decreased (Na) sodium concentrations than the non-mycorrhizal plants at more than one level of salinity in tomato and fenugreek plants while *Rhizhopagus intraradices* inoculated plants increased the concentrations of nitrogen (N), phosphorous (P), and potassium (K) (Hajiboland et al., 2010; Evelin et al., 2012). Also, plants colonized with *Funneliformis mosseae* maintained higher concentrations of N, P, and K and decreased the Na levels in cotton and citrus plants (Wu et al., 2013). Ample reports suggest the protective role of AMF in maintaining the ionic balance in plants under salt stress by increasing K^+^: Na^+^ ratios (Giri et al., 2007; Hajiboland et al., 2010; Evelin et al., 2012; Wu et al., 2013). Likewise, AMF inoculation of citrus plants induced significantly higher K uptake and significantly lower Na uptake than in control plants, indicating a preferential uptake of K against Na into the root xylem of stressed AM plants (Wu et al., 2010, 2013). In addition, AM symbiosis has been demarcated to increase stomatal conductance, transpiration and photosynthetic rates and water use efficiency in plants affected by salinity stress than non-mycorrhizal plants (Sheng et al., 2008;Evelin et al., 2012; Ruiz-Lozano et al., 2012; Shamshiri and Fattahi, 2016). Moreover, efficiency also varied among isolates of AMF irrespective of the individual host plant or geographical origin. Daei et al. (2009) reported that commonly used AMF species in experiments under saline conditions were *Glomus* spp., of which *Rhizophagus fasciculatus* appeared the most efficient in terms of plant performance and attenuation of detrimental effects posed by salinity. The effectiveness of inoculation of *R. fasciculatus* in alleviating salt stress and promoting plant growth in comparison to *R. intraradices* and *F. mosseae* can be attributed for efficacy variation toward stress tolerance among the fungal counterparts (Daei et al., 2009). Variation in plant growth stimulation by AMF has also been frequently reported under non-stressed conditions among isolates belonging to different species, as well as among isolates of the same species (van der Heijden et al., 1998; Munkvold et al., 2004; Jansa et al., 2008). Porras-Soriano et al. (2009) suggested that C_3_ plants, *Olea europaea* colonized with *F. mosseae*, *R. irregularis*, and *R. claroideum*, show a remarkable rise in salt tolerance due to N, P, and K uptake, precisely contributing plant growth and nutrient acquisition. They found that among AMF species, *F. mosseae* was the most efficient AMF in reducing the detrimental effects of salinity, and this effect was due to increased K uptake (Porras-Soriano et al., 2009). In addition, *Glomus deserticola* exhibited a higher symbiotic efficiency in C_3_ plants, *Lactuca sativa* compared to other *Glomus* sp. under saline condition. Zou and Wu (2011) proposed that the C_4_ plant *Poncirus trifoliata*, trifoliate orange seedlings when inoculated with five different AMF species (*Diversispora spurca*, *Claroideoglomus etunicatum*, *F. mosseae*, *G. versiforme*, and *Paraglomus occultum*) depict significant efficiency in symbiotic paradigm. In soils rich in salinity content, the order of efficiency is: *G. versiforme* > *D. spurca* > *F. mosseae* > *P. occultum* > *G. etunicatum*, which clearly indicate that mycorrhizal colonization, entry points, vesicles and arbuscles possess prominent roles for symbiotic implications. The plant parameters like plant height, stem diameter, shoot, root, and total dry weights were recorded with a characteristic increase along with a notable increase in root architecture comprising root tip numbers, length, surface area, projected area, and volume. *G. versiforme* depicted potentiating effects in combating salt stress of trifoliate orange compared to *C. etunicatum*. The above research perspectives are indicative of the fact that the compatibility of AMF and host plants is necessitated for mycorrhizal development for optimal outcomes. AMF can be used to alleviate salt stress of plants, but the resultant effect of symbiotic fungi is dependent on fungal species. There are a number of publications supporting the view that proline accumulation in response to salt stress is a positive indicator of stress perception (Vaidyanathan et al., 2003; Maiale et al., 2004) (Table 1). Investigations on osmoregulation by the AM symbiosis and AMF are relatively few and results are inconsistent. Proline content has been associated for stress amendments among AM plants and has been stressed for a focused role as an optimal factor in assessing AMF and salinity in plants (Sannazzaro et al., 2007; Echeverria et al., 2013). In parallel, AM symbiotic efficiency in plants shows convergent patterns depending on the plant species involved (Chandrasekaran et al., 2016).

**Table 1:** Level of proline accumulation influenced by salt stress in Arbuscular Mycorrhizal symbiotic plants.

Host species	Fungus species	Level of salinity (NaCl)	Duration (days)	Proline accumulation		Reference
				Aerial organs	Root	
*Capsicum annuum*	*R. intraradices*	50, 100, and 200 mM	56	Proline↑	Proline↑	Beltrano et al., 2013
*Pennisetum glaucum*	*R. fasiculatus*	100, 200, and 300 mM	90	Proline↓	Proline↑	Borde et al., 2011
*Gmelina arborea*	*R. fasiculatus*	100 and 200 mM	70	Proline↑	Proline↑	Dudhane et al., 2011
*Lotus tenuis*	*R. intraradices*	150 mM	5	Proline↓	Proline↓	Echeverria et al., 2013
*Ocimum basilicum*	*G. deserticola*	5 and 10 dS m^−1^	70	Proline↑	Proline↑	Elhindi et al., 2017
*Zea mays*	*R. intraradices* and *C. etunicatum*	66 and 100 mM	30	Proline↓	Proline↓	Estrada et al., 2013
*Trigonella foenum*	*R. intraradices*	50, 100, and 200 mM	7	Proline↓		Evelin et al., 2013
*Cicer arietinum*	*F. mosseae*	4, 6, and 8 dS m^−1^	80	Proline↑	Proline↓	Garg and Baher, 2013
*Solanum lycopersicum*	*R. intraradices*	5 and 10 dS m^−1^	84	Proline↑	Proline↑	Hajiboland et al., 2010
*Lactuca sativa*	*R. intraradices*	50 and 100 mM	30		Proline↑	Jahromi et al., 2008
*Vigna radiata*	*R. intraradices*	12.5 and 25 mM	62		Proline↑	Jindal et al., 1993
*Lactuva sativa*	*R. intraradices*	3, 4, and 5 g/kg	49		Proline↑	Ruiz-Lozano et al., 1996a
	*F. mosseae*				Proline↓	
*Lotus glaber*	*R. intraradices*	200 mM	28	Proline↓	Proline↑	Sannazzaro et al., 2007
*Ocimum basilicum*	*F. mosseae*	75 and 150 mM	4	Proline↑	Proline↑	Shekoofeh et al., 2012
	*R. intraradices*			Proline↑	Proline↑	

*Symbols↑, and ↓ means that AM symbiosis increased or decreased proline accumulation, respectively.*

## Amf Mediated Alleviation of Drought Stress

Drought stress abatement in plants has been contributed to morphological, anatomical, and cellular adaptations to elude either the stress or to augment its tolerance (Ruiz-Lozano, 2003). AM symbiosis is a prominent association that helps plants to survive with drought stress and amplify drought resistance (Ruiz-Lozano and Azcón, 1995; Augé, 2001; Ruiz-Lozano, 2003). AM possess the competence in absorbing soil nutrients and water for the plant partner owing to better plant growth and drought tolerance (Fahad et al., 2017). The mechanism involved in AMF-enhanced drought tolerance of host plants is poorly understood, although possible modes include direct water and nutrient uptake via extraradical hyphae, better root system architecture, enhancement of antioxidant defense systems, and greater osmotic adjustment. Earlier studies reported that a possible role of AMF hyphae in water uptake and transfer to the host plant. Hyphae with a diameter of 2–5 μm can penetrate soil pores that are inaccessible to root hairs (10–20 μm diameter) and absorb water owing to heightened symbiotic patterns through which non-mycorrhizal plants are readily able to access water potentials (Allen, 1982; Hardie, 1985; Ruiz-Lozano and Azcón, 1996). Extraradical AMF fungal mycelia have shown promising effects in contracting root surface area at ease and enhanced water uptake by host roots. Non-nutritional mechanisms proposed to explain this protection by AM symbiosis are accredited to plant hormones, increased gas exchange, and water status (Ruiz-Lozano and Azcón, 1995; Ruiz-Lozano et al., 1996a; Abbaspour et al., 2012), direct hyphal uptake of water from soil (Ruiz-Lozano and Azcón, 1995) and enhanced activities of ROS scavenging enzymes (Ruiz-Lozano et al., 1996b). In addition, AM symbiosis regulated a wide number of aquaporins in the host plant, comprising members of the different aquaporin subfamilies [NOD26-like intrinsic proteins (NIPs); plasma membrane intrinsic proteins (PIPs); tonoplast intrinsic proteins (TIPs)] (Bárzana et al., 2014; Quiroga et al., 2017). Most of these aquaporins can transport water and also other molecules (CO_2_, O_2_, silicon, boron, urea, or ammonia) of physiological importance for plant performance. They improve root hydraulic conductivity as well as the plant water status and tolerance under drought stress (Aroca et al., 2007; Bárzana et al., 2014; Chitarra et al., 2016). The regulation of these genes depends on the watering conditions and on the severity of the drought stress imposed (Aroca et al., 2007; Bárzana et al., 2014; Chitarra et al., 2016). Recently, *F. mosseae* inoculated plants showed differential transcriptional pattern of theses aquaporins genes, overexpression of *LeNIP3;1* was in AM plants, conversely, *LePIP1;1* and *LeTIP2;3* were down-regulated both in AM and non-AM plants (Chitarra et al., 2016). In addition, a number of studies have demonstrated that, during soil drying, mycorrhizal plants often maintain higher gas exchange rates than non-mycorrhizal plants of similar size and nutrient status (Bethlenfalvay et al., 1989; Ruiz-Lozano et al., 1996a,b; Augé, 2001; Augé et al., 2015). Duan et al. (1996) reported that mycorrhizal plants maintained higher stomatal conductance, transpiration rate and shoot water than non-mycorrhizal plants. Duan et al. (1996) have suggested that AMF probably increase the ability of the root system to scavenge water in the dried soil, resulting in less strain on foliage, and hence higher stomatal conductance and shoot water content at a particular soil water potential. In the absence of a clear plant-based explanation for mycorrhizal influence on stomatal conductance and other leaf water relations, symbiosis largely affects water retention properties and water uptake in soils marking a prospective consequence for AM and non-AM plants inoculation strategies (Augé et al., 2001). According to Ruiz-Lozano et al. (1995) the detrimental effects of drought were not related to decreases in photosynthesis or water use efficiency. They proposed that the differences in P and K acquisition, transpiration, and stomatal conductance were related to the mycorrhizal efficiencies of the different fungi. Differential properties of fungi on assuaging stress can be potent characteristic reliable on physiological processes having little influence of the host plant’s nutrient uptake. Studies pertaining to drought resistance through fungus-host plant interactions pose a special arena of research due to unavailability of water under most circumstances. Ruiz-Lozano et al. (1995) also reported that *G. deserticola* reduced drought stress comprehensively by 9% when compared to *G. etunicatum* by 17% indicating plant growth properties. Nevertheless, the colonization efficacy of both the isolates comparatively did not exhibit variations in nutrient contents that are essential for plant growth. Nevertheless, CO_2_ exchange rates and water use efficiency values were significantly lower in plants colonized by *G. etunicatum* than in plants colonized by *G. deserticola*, *G. occultum* inoculated plants reflect a drastic decline in shoot biomass by 70% when compared to 9% activity manifested by *G. deserticola*. They suggested that effectiveness of *Glomus* species (*G. deserticola* > *R. fasciculatus* > *F. mosseae* > *G. etunicatum* > *R. intraradices* > *G. caledonium* > *G. ocultum*) in for a decline in shoot dry weight as a result of stimulating increased tolerance to water stress. Moreover, changes in increased CO_2_ assimilation, stomatal conductance (Augé and Duan, 1991) and transpiration have been reported to be mechanisms by which AM fungus-colonized plants increase drought resistance. In their studies, they could not find any close relationship between AMF-colonized plants and resistance to drought. Hence insights into microbiome population of host plants and interaction assessment between AMF could also become a promising area of research in the near future.

Under drought stress conditions, adaptive mechanisms by host plants when colonized by *F. mosseae* were ascribed for better photosynthetic activity and proline accumulation indicating strong evidence for positive effects of symbiosis (Azcón et al., 1996). However, in the same study, several nutrients (N, P, and Mg) and the proline content increased in *R. fasciculatus* colonized plants showing low photosynthetic activity in relation to water stress. Among macronutrients, K being a cationic solute has a pivotal responsibility in increased stomatal function for lessening water stress in host plants under diverse conditions ranging from low to bulk availability of water status in leaves of a plant. Hence, K content and stress tolerance are positively corroborated in *Glomus*-infected plants. The above phenomenon correlates conclusively wherein, *F. mosseae* colonization subjected to water stress was observed with enhanced K uptake by plants (Azcón et al., 1996). Further confirmatory of the fact *F. mosseae* colonized plants signify a plethora of adaptive properties resulting in elevated plant growth through augmented photosynthetic activity and proline accumulation (Azcón et al., 1996). This affirms that proline accumulation contributes to plant growth under severe stress indirectly. On the basis of these facts, no clear correlations between nutritive and/or physiological abilities and drought resistance can be confirmed in mycorrhizal plants. The molecular mechanisms underlying the contributory roles will take a progressive time in dissecting the event. We provide initial cohesive roles for proline accumulation and AMF symbiosis in host plant growth based on combinatorial perspectives of plant growth.

Keeping a view of the proline accumulation and AMF symbiosis in plant growth, interdisciplinary research which would address the necessary roles for the relationship between host plants and water stress with a view for AMF symbiosis in a direct manner or complex roles for endophytes in plants networking with AMF creates an intriguing thought in interpreting symbiosis. Geographical information system analysis with interpolation studies using remote sensing tools will provide deep insights into region-specific differences in geographical isolates (Bethlenfalvay et al., 1989) for symbiosis conclusion. Furthermore, proline accumulation will shed a glimpse on the above-said study parameters in establishing AMF-Plant symbiosis and endophytic population in the elucidation of positive roles for Photosynthetic machinery, stress tolerance, nutrient uptake finally leading to plant growth and increased productivity. We also hypothesize that endophytic population of host plants or plant microbiome analysis for every plant will yield promising results highlighting a revolutionary food supply all throughout the world sustainably. Knowledge concerning specific relationships between plants and fungi is important for successful utilization of AM fungi under particular conditions. In general, proline accumulation positively correlates with drought tolerance of AMF-colonized plants. Furthermore, proline accumulation was found to vary quantitatively when the host plant is colonized by AMF (Evelin et al., 2009). Perception for proline accumulation in response to stress is a good indicator of a higher stress has also been advocated earlier (Delauney and Verma, 1993; Kishor et al., 2005), it might serve as a parameter to evaluate the effects of AMF in depicting salinity and drought on plants (Sannazzaro et al., 2007; Abbaspour et al., 2012; Echeverria et al., 2013) (Table 2).

## Proline Biosynthesis and Accumulation

Biosynthesis of proline has been linked to glutamate or the ornithine pathway. Especially, connected to the oxidative pentose phosphate pathway and glutamate-glutamine metabolism (Verslues and Sharma, 2010). Proline is synthesized via glutamate pathway in the cytoplasm in which, glutamate converted into 1-pyrroline-5-carboxylate (P5C) by Δ 1-pyrroline-5-carboxylate synthetase (P5CS), which is converted into proline by Δ 1-pyrroline-5-carboxylate reductase (P5CR). On the other hand, the ornithine pathway corresponds to production of proline from ornithine in the mitochondrion. Ornithine is transaminated by ornithine-δ aminotransferase (OAT) to form P5C and glutamate-semialdehyde, and transformed to proline. Moreover, ProDH, a key enzyme in proline catabolism catabolized proline into P5C (Trovato et al., 2008). The resultant bioproducts are two proline synthetases, P5CS, and OAT. This concept affirms that the proline catabolic enzyme ProDH is the key regulator in the cumulative proline accumulation in plants (Szabados and Savoure, 2009).

## Role of Proline in Salinity Tolerance

Proline has been identified as an optimistic indicator in perceiving stress under increased salinity situations by various researchers (Subramanian and Charest, 1999; Maiale et al., 2004; Sannazzaro et al., 2007). Further, the salinity conditions can also be related to water use efficiency and availability for plants (Evelin et al., 2009; Hayat et al., 2012; Porcel et al., 2012). AMF inoculation on plants shows altered efficacies in combating salinity stress among mycorrhizal plants and proline content act as a dynamic factor to assess salinity consequences (Sannazzaro et al., 2007; Echeverria et al., 2013). Alternatively, there are also reports which show that AMF inoculation considerably decreased proline accumulation (Wu and Xia, 2006; Sheng et al., 2008; Borde et al., 2011; Echeverria et al., 2013) while sufficient data demonstrate the increase (Jindal et al., 1993; Jahromi et al., 2008; Elhindi et al., 2017). On the contrary, some studies also indicate a null effect on proline accumulation (Dudhane et al., 2011; Estrada et al., 2013) (Table 1). According to Ruiz-Lozano et al. (1996a), mycorrhizal plants are lower in proline accumulation (except *F. mosseae* colonized plants) than those of non-mycorrhizal plants. However, the same study also reported that accumulation of proline did not increase significantly at different levels of salinity in *Glomus deserticola* colonized plants. But, in the case of *Rhizophagus fasciculatus*, the increase in proline accumulation was significant at the highest level of salinity (5 g NaCl kg^−1^). Proline accumulation was also found to be increased in control plants with increasing salinity. Dudhane et al. (2011) suggest that proline accumulation in both non-mycorrhizal and mycorrhizal plants increased significantly by increasing salinity. Rabie and Almadini (2005) also reported increasing salinity level resulted in an increase of the proline concentrations in both AM plants and non-AM plants, when compared to AM plants, non-AM plants had a higher proline concentration especially at high level of salinity. *F. mosseae* colonized *Ocimum basilicum* plants showed a significant increase in proline content compared to control plants (Shekoofeh et al., 2012). However, plants inoculated with *R. intraradices* depict the rise in proline prominently under low levels of salinity than control plant which show a considerable increase in the high level of salinity (Wu et al., 2016). Moreover, Hashem et al. (2015) reported that proline accumulation in mycorrhizal (*F. mosseae*, *R. intraradices*, and *C. etunicatum*) plants have a short and effective outcome on the osmoregulation of the plants.

Wherein, *R. intraradices* inoculated plants show less contrast in proline accumulation symbolizing mycorrhizal plants are less affected by salinity stress due to proline levels (Echeverria et al., 2013). Dosage compensation studies encompassing minimal mycorrhizal treatments [5 g (75 spores)] than [10 g (150 spores)] and [20 g dosages (300 spores)] increased proline accumulation which specifies mycorrhizal inoculation on tomato plants at a nominal inoculation shows considerable activity. This fact provides us with an evidence for socio-economic benefits of AMF in small scale (Damaiyanti et al., 2015). Recently, Elhindi et al. (2017) observed that the proline accumulation decreased significantly in AM plants under salt stress. The decrease in proline accumulation in *G. deserticola* inoculated *O. basilicum* plants present lucid conclusion that mycorrhizal plants evaded stress due to proline accumulation. Meta-analysis for efficiency among AMF species, *R. fasciculatus* and *F. mossea* also affirm that AMF symbiosis significantly showed proline accumulation subjected to salt stress. Relatively, Proline content was lessened in case of *R. intraradices* alone, which demonstrate AMF symbiosis and proline accumulation can have alternate solutions for an inclusive linkage for symbiosis and plant growth (Chandrasekaran et al., 2014). Their studies also suggested that among plant species, AM associated *Zea mays* showed a significant decrease in proline accumulation whereas some other AM associated plant species such as *Gmelina arborea* followed by *Cicer arietinum* and *Allium sativum* showed an increased level of proline accumulation. In comparison on plant functional groups showed that trees showed increased proline accumulation whereas herbs showed decreased proline accumulation in Am symbiotic plants under salt stress.

Recently, it was confirmed from meta-analysis studies that variation in proline accumulation among AMF species in C_3_ and C_4_ plant species. Proline accumulation inoculated with *R. fasciculatus* plants were observed considerably with high proline content than with *R. irregularis* and *F. mosseae* inoculated plants. Further, there was not a definitive conclusion whether C_3_ or C_4_ plants had higher proline accumulation. These variations upon analysis also show that AMF symbiosis is not restricted to a particular plant, but can be rationalized upon repetitive field trials and mock control experiments in identifying a specificity of AMF symbiosis in a wide angle of research areas, precisely a 360° approach analysis for an explicit solution. C_4_ monocotyledonous plants had a prominent rise in proline accumulation than C_3_ dicotyledonous plants. But C_3_ dicotyledonous plants were considered superior compared to proline accumulation than non-mycorrhizal counterparts. The study after an extensive array of analysis revealed that amongst various predictor variables, AMF as an identity for salt stress to subside salt stress was conclusive (Chandrasekaran et al., 2016). Numerous research report that non-mycorrhizal plants accumulated proline higher than AM plants at different salinity levels, suggesting that proline accumulation in mycorrhizal plants could act as an indicator of stress (Borde et al., 2011; Damaiyanti et al., 2015). The wide literature analysis provides us with a strong notion that AMF symbiosis induced benefits and proline accumulation are evidently the possible modality in concentrating toward productive contribution with reference to plant growth and technology up-gradation for a lucid research with proline and AMF symbiosis.

## Role of Proline in Drought Tolerance

Counteracting mechanisms by mycorrhizal plants against osmotic stress caused by drought is mediated through biochemical changes that assist in the escalated secretion of osmolytes like proline. The proline thus accumulated helps in minimizing osmotic potential in turn leaf water potential which renders the host plants to sustain the photosynthetic apparatus by retaining elevated organ hydration and turgor pressure maintenance (Ruiz-Lozano et al., 1995; Wang et al., 2004; Kandowangko et al., 2009). Constructive correlation among AMF mediated drought tolerance in host plants and proline accumulation has been addressed significantly by many researchers (Ruiz-Lozano et al., 1995; Wang et al., 2004; Kandowangko et al., 2009; Yooyongwech et al., 2013). Proline accumulation levels are divergent with varying degrees of osmotic alterations owing to the fact that AMF symbiosis and osmotic regulation. The AM plants accumulated significantly higher level of proline upon exposure to drought stress when compared with the non-AM plants. A higher content of proline in AM plants suggests that the plants inoculated AMF might have a better capacity for osmotic adjustment relative to the non-AM plants in the presence of drought stress. Similar findings were reported for other species, such as *Antirhinum majus* (Asrar et al., 2012), *Erythrina variegata* (Manoharan et al., 2010), *Cyclobalanopsis glauca* (Zhang et al., 2014), and *Ocimum gratissimum* (Hazzoumi et al., 2015). In disparity, *Oryza sativa* associated with AMF exposed to drought also progressively possessed elevated proline (Hu et al., 1992) and *Macadamia tetraphylla* (Roosens et al., 1998). Ruiz-Lozano et al. (1995) found that droughted salad plants inoculated with *G. deserticola* showed high proline content ∼119.6 nmol/g fresh weight than non-mycorrhizal associations which had a minimal amount of proline, about 16.2 nmol/g/fresh weight. In a similar study, *F. mosseae* and *R. fasciculatus* inoculated plants redressed high proline accumulation than non-mycorrhizal plants which evidently show the adaptive mechanisms in efficiently combating drought resistance (Azcón et al., 1996). Previously, analogous results were substantiated when mycorrhizal plants show a high incidence of proline under drought stress in *Poncirus trifoliata* plants (Lu et al., 2007). AMF inoculated “H2” cultivar of *Macadamia tetraphylla* plants when experimented with water discrepancy showed 40 μmol g^−1^ fresh weights of proline as compared to those of non-mycorrhizal plants which showed ∼5 μmol g^−1^ fresh weights of proline (Yooyongwech et al., 2013). Similarly, high proline accumulation was perceived in drought plants grown in symbiosis with AMF, *F. mosseae* compared to control (Chitarra et al., 2016). High proline content in AMF inoculated plants play a vital function in sugar modulation, increasing water content in leaves, enhanced photosynthetic machinery which collectively yields excellent growth under drought stress (Foyer et al., 2017). AMF hence play multiple roles in plant productivity, protection, and crop improvement strategies. AMF has been a classical modality in increasing plant efficiency and efficacy in providing farmers with a huge profit in most of the plant associations. The recent era has witnessed a revolution in organic agriculture and AMF play a pivotal role in superior strategies for crop improvement. This review will possibly dissect their roles at physiological, biochemical and molecular levels. Further, degradation of proline yields high energy, about 30 ATP equivalents per molecule (Liang et al., 2013). Hence, AMF also can be linked as a rich energy supplement in terms of proline accumulation. This fact already has been documented as proline in AM pretreated plants upholds energy levels and support plant growth under severe osmotic stress (Hayat et al., 2012). On the contrary, in several studies, a decrease in proline accumulation was witnessed in mycorrhizal plants compared to those non-mycorrhizal equivalent (Hong et al., 2000; Aroca et al., 2008; Ruiz-Sánchez et al., 2010; Fan and Liu, 2011; Abbaspour et al., 2012) (Table 2), which further adds to the actuality that symbiotic association with AMF enhanced the host plant tolerance to drought stress.

**Table 2:** Level of proline accumulation influenced by drought stress in Arbuscular Mycorrhizal symbiotic plants.

Host species	Fungus species	Level of drought	Duration (days)	Proline accumulation		Reference
				Aerial organs	Root	
*Pistacia vera*	*C. etunicatum*	50% water holding capacity	30	Proline↓	Proline↓	Abbaspour et al., 2012
*Antirhinum majus*	*G. deserticola*	Water content (−0.14, −0.38, and −0.55 MPa)	70	Proline↓		Asrar et al., 2012
*Solanum lycopersicum*	*F. mosseae* and *R. intraradices*	Water irrigation was withheld about 3 weeks	20	Proline↑	Proline↑	Chitarra et al., 2016
*Knautia arvensis*	*Glomus* sp.	Water regimes 2555% field capacity	84		Proline↓	Doubková et al., 2013
*Poncirus trifoliata*	*F. mosseae*	With holding water for 3 days	4	Proline↑	Proline↑	Fan and Liu, 2011
*Dracaena fragrans*	*R. manihotis*	30% water availability	55	Proline↑	Proline↑	Kandowangko et al., 2009
*Erythrina variegata*	*R. intraradices*	Leaf water potential (−1.20, −2.20, and −3.40 MPa)	60	Proline↓	Proline↓	Manoharan et al., 2010
*Citrus tangerine*	*G. versiforme*	55% relative water content	80	Proline↓	Proline↓	Wu et al., 2006
*Poncirus trifoliata*	*G. versiforme*	Soil water content (−0.09 and −0.40 MPa)	80	Proline↓	Proline↓	Wu et al., 2007
*Poncirus trifoliata*	*F. mosseae* and *Paraglomus occultum*	50% Water holding capacity	71	Proline↓		Wu et al., 2017
*Poncirus trifoliata*	*F. mosseae*	57% water holding capacity	80	Proline↓	Proline↓	Zou et al., 2013

*Symbols ↑, and ↓ means that AM symbiosis increased or decreased proline accumulation, respectively.*

It was also earlier recognized that proline accumulation under drought stress is minimized in aerial organs of mycorrhizal plants than in non-mycorrhizal plants of *Citrus tangerine* (Abbaspour et al., 2012), *Erythrina variegata* (Doubková et al., 2013), *Macadamia tetraphylla* (Kandowangko et al., 2009), *Pistacia vera* (Manoharan et al., 2010), and *Antirhinum majus* (Abbaspour et al., 2011). AMF colonization with less proline accumulation face reduced threat under drought. Hence proline can also be regarded as an osmoprotectant apart from inducing plant defense mechanisms as a result of osmolyte balance. On contrary, in spite of ample water availability conditions, proline content was remarkably reduced under *F. mosseae* colonization in *P. trifoliata* seedlings (Zou et al., 2013), which provides the determinative conclusion that plants colonized by AMF comparatively abate injury due to drought and evade stress adversities with less proline content. However, varied accumulation of proline in distinct plant parts have been a varied phenomenon. Mycorrhization in *Glycine max* resulted in increased proline accumulation in roots but not in shoots under drought stress (Asrar et al., 2012).

High proline content in soybean associated mycorrhizal roots than in shoots in severe drought (Porcel and Ruiz-Lozano, 2004) show a positive association of mycorrhiza with different parts of the same plant under stress. This varied accumulation can have multiple effects upon the plant growth and photosynthesis which opens up several avenues of research. Earlier Pinior et al. (2005), suggested for a thorough analysis of below and aerial organs under drought stress in the near future. We also suggest for dissection studies in individual parts aiming to study photosynthesis machinery and proline for wider prospects with regard to AMF. These results may be recognized as effective strategies to combat better drought resistance of AM plants and avoid injury under drought (Porcel et al., 2004; Wu et al., 2006; Zou et al., 2013). It can be foreseen that proline accumulation might be used as an appropriate marker to assess AMF and plant symbiosis with respect to drought and osmotic stress. Further, when suitable studies are ample we can also propose that AMF will act as a potent factor to address almost all stress not only drought. Proline accumulation in AM plants also is remarkably regarded as a suitable indicator under lower content responsible for surpassing maximal injury caused by drought (Zou et al., 2013). Further, Proline also appropriates drought tolerance in host plants (Augé and Moore, 2005). Proline accumulation in less tolerant plants has also been linked to a progressive agent of tissue injury as an accompanying consequence of NaCl exposure (Ruiz-Lozano et al., 2012). The same research group also demonstrated that non-mycorrhizal plants accumulated more proline in their shoots than AM plants especially lettuce influenced by drought. These results also are asserting a point of intricacy involving AM symbiosis for altered plant responses (Ruiz-Lozano et al., 2011). Comparatively, AM lettuce plants accumulation of proline was elucidated with the verity that AM plants employ proline accumulation to manage low water potentials in dry soils. Under favorable conditions, AM plants maintain water potential gradient that enriches water uptake by plant roots (Porcel and Ruiz-Lozano, 2004).

It has been more regarded that AMF symbiosis and benefits are overlooked in certain circumstances. But, to be precise, AM plants provide enormous benefits to the host than NM plants acting as an indicator of injury (Augé et al., 2014). Plants which are supplied with ample water for growth possess less proline content, compared to plants under drought stress. The research also revealed that *G. occultum*, *R. fasciculatus*, and *F. mosseae* enhanced proline production by 73, 89, and 107% when compared to *G. deserticola* and *G. etunicatum* which escalated proline accumulation by only 50% for relieving drought stress for a similar outcome. Drought tolerance is well-signified by low proline content and thus is regarded as an indicator for resistance capability of a host plant. The geographical location and the presence of copious beneficiary fungal population predominantly determines the host response under initial conditions. The host response also invariably depends on multiple factors ranging from mineral uptake efficiency, physiological interactions between the symbionts and host, as fungal protective efficiency in a physiological relationship depends on the amount of nutrient uptake thereby finally yielding a plant growth which provides optimal benefits for the mankind.

## Molecular Changes in *P5CS* in AMF Inoculated Plants

Although the role of AMF in plant osmotic stress tolerance has been well-reported for several plants-AMF studies, the fundamental molecular interactions are still to be revealed. In this regard, several studies have been put forth for enhancing the AMF and proline accumulation. In a similar study, four genes namely *KvP5CS1*, *KvOAT*, *KvPDH*, and *KvProT* from *Kosteletzkya virginica* seedlings under salt stress were cloned and expression profiling revealed that *KvP5CS1* and *KvOAT* resulted in proline accumulation in leaves were up-regulated, and *KvPDH* was down-regulated indicating proline accumulation before 12 h under salt stress (Wang et al., 2015). All the molecular revelations show that there is a close resemblance of activity profiling and expression patterns corresponding to proline and osmotic stress tolerance (Maiale et al., 2004; Kishor et al., 2005; Ribarits et al., 2007). Expression of *Lsp5cs* gene was found up-regulated regulated in non-mycorrhizal *Lactuca sativa* plants than in *R. intraradices* colonized plants under low salinity profiles. On the contrary, *Lsp5cs* gene was up-regulated in non-mycorrhizal *Lactuca sativa* plants than in *R. intraradices.* But salinity rise didn’t show significant *Lsp5cs* gene expression and proline accumulation in both mycorrhizal and non-mycorrhizal plants (Jahromi et al., 2008). In contrast, Garg and Baher (2013) suggested that the association of chickpea roots with *F. mosseae* increased proline content had a direct correlation with increased Na^+^ content in leaves. The quantitative analysis of proline revealed significantly higher levels of this amino acid at 8 dS m^−1^ than at 6 and 4 dS m^−1^ in leaves when compared with roots. In addition, salinity stress led to a higher increase in the activity of enzymes of the proline biosynthetic pathway in leaves when compared with roots, with P5CS and GDH (glutamine dehydrogenase). On the other hand, salinity induced a remarkable reduction in ProDH, suggesting that accumulation of proline was the result of concomitant inactivation of ProDH, thus negatively correlated with sodium content in leaves as well as in roots (Garg and Baher, 2013). Transcriptome analysis using real-time reverse transcriptase by Abo-Doma et al. (2011) indicated that mycorrhiza treatment resulted in an increase in the gene expression of *P5CS* on the transcription level. Hence, there is a profound gap to be abridged and coherent results regarding expression patterns at a molecular level that can properly be utilized to link and demarcate a lineage of genes specific for proline accumulation, osmotic stress and delinking mycorrhizal and non-mycorrhizal counterparts with proline accumulation.

Proline levels under drought stress is consequently dependent upon escalated glutamate synthetic pathway and reduced proline catabolism (Verslues and Sharma, 2010). Moreover, Wu et al. (2017) reported that AMF seedlings showed significantly lower P5CR and P5CS activity but substantially higher ProDH activity than non-AMF seedlings, under drought conditions. Zou et al. (2013) also suggested that drought stress induced higher proline concentrations accompanied by an increase of P5CR and P5CS activity, a decrease of OAT activity and no difference of ProDH. This means that a decrease in proline accumulation in AMF seedlings is potentially associated with an AMF-modulated decrease of glutamate synthetic pathways but not the ornithine synthetic pathway of proline and an increase of proline catabolism.

An interesting fact observed was mycorrhizal trifoliate orange upon drought stress showed greater root adaptation morphology and increase in phytohormones when inoculated with *Diversispora versiformis* showing greater concentrations of indole-3-acetic acid, methyl jasmonate, nitric oxide, and calmodulin in roots (Zou et al., 2017). These results further leave us with more intriguing research that can be foreseen in the next years of extensive analysis. The present review can act as a launchpad for researchers with unanswerable facts in the field of AMF and proline accumulation. Genes for P5CS involved in drought stress in *Glycine max* and *Lactuca sativa* plants showed contrasting results. Inoculation with *Bradyrhizobium japonicum* clearly indicates that induction of *p5cs* gene cannot be regarded as a mechanism by AMF in alleviating drought stress (Porcel et al., 2004). Further studies are thus needed to understand these discrepancies if a net proline accumulation relates to the activity of proline synthetases and/or catabolic enzymes in AM plants under drought. These research arenas almost give us a complex situation to make way for further detailed description and analysis of AMF and proline accumulation. However, the effect of AMF on proline metabolism in the present review will act as a launchpad for further futuristic research pertaining to AMF, Proline accumulation, Endophytes of host plants and their interaction in augmenting plant growth. We believe that interdiscplinary area of research pertaining the present review will foresee explicit advancements in the field of AMF research.

## Conclusion and Future Perspectives

The review comprehensively compiles significant correlations and limitations associated with plant stress tolerance and evasion mechanisms. Proline has every possibility of consideration as an indicator and potential marker for possible injury by osmotic stress. However, numerous questions related to proline remain still unanswered. Degradation and toxicity of proline pose potential threats in addressing proline accumulation and osmotic stress evasion particularly salinity and drought stress. Investigation of the proline genes in stress evasion has to be addressed to their genetics, regulation, and evolution. Further, Proline accumulation and stress tolerance needs to be unraveled with protein-protein interaction perspectives and also interacting strategies concerned to DNA and RNA involved in proline accumulation with regard to osmotic stress both in symbiotic mycorrhizal and soil associated mycorrhiza. Further, mycorrhizal and non- mycorrhizal interacting host plants and proline expression profiling will foresee unequivocal results in the arena of AMF and proline accumulation. In the case of redundant gene families, studies on multiple proline mutants will be needed to characterize their role in stress tolerance. Particularly, more studies are required to understand the expression of *p5cs* genes in the proline biosynthesis of AMF inoculated plants. Therefore, metabolomics and stress gene expression profiling will help to identify specific targets of AMF mediated proline accumulation in stress tolerance, thus opening new paths for the development of more stress tolerance crops and the development of sustainable agricultural practices. The present review will provide more research avenues concerned with AMF and proline accumulation not only in osmotic stress perspectives but also a rationalized conceptual framework for future research in other stresses apprehensive in the AMF and proline accumulation.

## Author Contributions

SC and MC conceived the idea. MC and MP wrote the main manuscript text. SC and MC prepared the manuscript. SC, MC and MP revised the manuscript. SC, MC and MP revised the text at different stages of the writing process and read and approved the current manuscript.

## Conflict of Interest Statement

The authors declare that the research was conducted in the absence of any commercial or financial relationships that could be construed as a potential conflict of interest.
